# Effectiveness of Sitagliptin and Empagliflozin Combination Therapy in a Patient With Charcot-Marie-Tooth Disease and Comorbid Diabetes Mellitus: A Case Report

**DOI:** 10.7759/cureus.89954

**Published:** 2025-08-12

**Authors:** Makoto Wada, Yuri Mizuno, Amika Kajiyama, Shuji Toriumi, Takahide Hashimoto, Naoki Ohtake, Masaya Yamaga, Tomohiko Yoshida, Hiroyuki Murai, Minoru Takemoto

**Affiliations:** 1 Department of Diabetes, Metabolism and Endocrinology, International University of Health and Welfare, Narita Hospital, Narita, JPN; 2 Department of Endocrinology, Hematology and Gerontology, Chiba University Graduate School of Medicine, Chiba, JPN; 3 Department of Neurology, International University of Health and Welfare, Narita Hospital, Narita, JPN

**Keywords:** charcot-marie-tooth disease, diabetes, dpp-4 inhibitor, sarcopenia, sglt2 inhibitor

## Abstract

This case report describes a 61-year-old male with Charcot-Marie-Tooth disease type 1A (CMT1A) who developed poorly controlled type 2 diabetes mellitus. The patient, with a history of CMT1A, was admitted for preoperative glycemic management prior to lumbar spinal stenosis surgery, exhibiting an HbA1c of 8.1%. Treatment with metformin had been insufficient. Considering potential insulin resistance due to decreased skeletal muscle mass from CMT and reduced mobility, a combination therapy of the DPP-4 inhibitor sitagliptin and the SGLT2 inhibitor empagliflozin was initiated post-surgery, alongside diet and exercise. Notably, this combination effectively improved glycemic control without reducing skeletal muscle mass. This suggests that the combination of a DPP-4 inhibitor and an SGLT2 inhibitor may be a viable therapeutic option for managing diabetes mellitus in patients with CMT, warranting further investigation in larger studies due to the rarity of this comorbidity. Long-term glycemic control is crucial for maintaining activities of daily living (ADLs) and quality of life (QOL) in these patients.

## Introduction

Charcot-Marie-Tooth disease (CMT) is the most common inherited peripheral neuropathy characterized by progressive motor and sensory nerve dysfunction, including distinctive atrophy of the calf muscles [1]. Its prevalence is reportedly one in 2500 [2]. The age of onset, severity, and specific characteristics of motor impairment vary depending on the subtype [3,4]. CMT1 is characterized by muscle weakness and sensory disturbances mainly in the distal lower extremities, accompanied by gait disturbances. Among its subtypes, CMT1A, caused by duplication of the PMP22 gene, is the most prevalent form of CMT1. The co-occurrence of CMT and diabetes mellitus is rare, and there are few reports on effective treatments for diabetes mellitus [5]. Here, we report a case of CMT complicated by diabetes mellitus and discuss treatment options.

## Case presentation

The patient was a 61-year-old man. He frequently experienced falls during childhood and exhibited poor motor performance, particularly in athletic activities. From the age of 20 years, symptoms such as weakness and numbness in the lower limbs appeared and progressed slowly. Eight years before visiting our department, he had been diagnosed with diabetes mellitus, hypertension, and liver dysfunction during a medical check-up. Two years prior to his admission, he began to experience gait instability on uneven surfaces and frequent falls. He was subsequently referred to the Neurology Department of our hospital. He was diagnosed with CMT type 1A based on the findings of muscle weakness and atrophy in the extremities, sensory disturbances, advanced peripheral neuropathy on nerve conduction velocity testing, and duplication of the PMP22 gene on genetic testing. He was scheduled for surgery in the Orthopedic Surgery Department of our hospital for lumbar spinal stenosis. Patients with CMT are at increased risk of developing spinal degenerative changes, including lumbar spinal stenosis, possibly due to altered gait biomechanics, chronic muscle weakness, and postural imbalances. However, preoperative testing revealed poor glycemic control with an HbA1c level of 8.1%, and he was admitted to our department for preoperative glycemic management.

His medical history included hypertension, lung cancer, and hepatocellular carcinoma; however, the recurrence of lung cancer and hepatocellular carcinoma has not been reported.

For diabetes mellitus, he was taking metformin (biguanide) 1000 mg. For hypertension, he was prescribed enalapril 10 mg, amlodipine 10 mg, and trichlormethiazide 4 mg. For neuropathic pain and analgesia, he was taking pregabalin 150 mg and loxoprofen 180 mg. For gastrointestinal protection, he was using rebamipide 300 mg and albumin tannate 13.5 g. As nutritional and vitamin supplements, he received fursultiamine 75 mg and mecobalamin 250 μg. For psychiatric symptoms, duloxetine 20 mg and venlafaxine 37.5 mg were prescribed. In addition, he was taking Lanser 1 mg.

His family history included diabetes mellitus, hypertension, and myocardial infarction in his father, cerebral infarction in his mother, and suspected CMT in his brother, although genetic analyses have not yet been performed on the sibling. His social history included drinking 300 ml of shochu, a traditional Japanese distilled spirit, per day until four months before admission and a smoking history of 40 cigarettes per day from the age of 20 to 60 years, but he had quit smoking one year before admission. His physical findings on admission were as follows: height, 161 cm; weight, 69.2 kg; and body mass index (BMI), 26.7. No abnormalities were observed in the heart, lungs, or abdomen; however, atrophy of the lower limb muscles was observed. The circumference of the right thigh was 46.2 cm, and the left thigh was 46 cm (normal value: 48 cm or more for men). The circumference of the right and left lower legs was 37.7 cm and 37.2 cm, respectively (measured at the maximum calf girth; normal value for men: ≥33 cm). Although these values exceed the reference range, the findings are considered to reflect subcutaneous fat accumulation rather than preserved muscle mass, as evident from the marked muscle atrophy observed on physical examination and supported by electrophysiological findings. Abnormal sensation was present in both lower limbs, and the Achilles tendon reflex was absent bilaterally. Monofilament testing was positive in both feet, indicating reduced pressure sensation consistent with peripheral neuropathy. Mild atrophy was observed in the first dorsal interosseous and hypothenar muscles of both the upper limbs. In both lower limbs, the muscle strength of the tibialis anterior and gastrocnemius was reduced (manual muscle testing grade 3-4), and muscle atrophy was observed. By contrast, no obvious abnormalities were observed in the cranial nervous system. The weight that could be lifted with the upper limbs was within 10 kg on both the left and right sides, the distance he could walk was 100 m, and he could not stand on one leg on either side, as shown in Table 1.

**Table 1:** Evaluation of muscle strength in the present case.

Function	Right	Left	Description
Upper limb lifting	Up to 10 kg	Up to 10 kg	Maximum load lifted
One-leg standing	Unable	Unable	Could not maintain position
Independent standing	–	–	Unable to maintain standing position
Maximum walking distance	–	–	100 meters

Nerve conduction velocity testing revealed mixed peripheral neuropathy in the upper limbs; however, this could not be measured in the lower limbs.

Table 2 shows the laboratory data of the patients at the time of examination.

**Table 2:** Laboratory data. CBC: complete blood count, TP: total protein, Alb: albumin, AST: aspartate aminotransferase, ALT: alanine aminotransferase, g-GT: gamma glutamyl transferase, T-Bil: total bilirubin, UA: uric acid, CPR: C-reactive protein, TNF-α: tumor necrosis factor-alpha, IL-6: interleukin-6, BUN: blood urea nitrogen, IRI: immune reactive insulin, HOMA-IR: homeostatic model assessment insulin resistance, TG: triglyceride, LDL-C: low-density lipoprotein cholesterol, HDL-C: high-density lipoprotein cholesterol, VB12: vitamin B 12, TSH: thyroid-stimulating hormone, FT4: free thyroxine Reference values for laboratory test are based on facility standards at our institution.

CBC	Reference range	On the current presentation		Reference range	On the current presentation
WBC (x10^2^/μL)	33-86	28	CRP (mg/dL)	0.00-0.14	0.18
RBC (x10^4^/μL)	435-555	332	Leptin (ng/dL)	0.6-8.9	8.3
Hb (g/dL)	13.7-16.8	12	TNF-α(pg/mL)	2.27-11.2	0.72
Ht (%)	40.7-50.1	34	IL-6 (pg/mL)	<7.0	2.9
Plt (x10^3^/μL)	158-348	53	BUN (mg/dL)	8-20	15.3
Biochemistry			Cre (mg/dL)	0.65-1.07	0.61
TP (g/dL)	6.6-8.1	6.5	Glucose (mg/dL)	73-109	97
Alb(g/dL)	4.1-5.1	3.6	HbA1c (%)	4.9-6.0	8.1
AST (U/L)	13-30	32	IRI (mU/mL)	1.2-9.0	17
ALT (U/L)	10-42	23	HOMA-IR	≦1.6	4.1
g-GT (U/L)	13-64	268	C-peptide (ng/mL)	0.67-2.48	2.04
T-bil (mg/dL)	0.4-1.5	0.8	TG (mg/dL)	35-149	139
UA (mg/dL)	<7.0	7	LDL-C (mg/dL)	≦139	104
Na (mmol/L)	138-145	138	HDL-C (mg/dL)	≧40	39
K (mmol/L)	3.6-4.8	4.4	VB12 (pg/mL)	180-914	321
Cl (mmol/L)	101-108	104	Folic acid (pg/mL)	≧4.0	5.8
Ca (mg/dL)	8.8-10.1	9.6	TSH (μIU/mL)	0.35-4.94	2.59
P (mg/dL)	2.7-4.6	3.7	FT4 (ng/mL)	0.7-1.48	1.2

**Figure 1:**
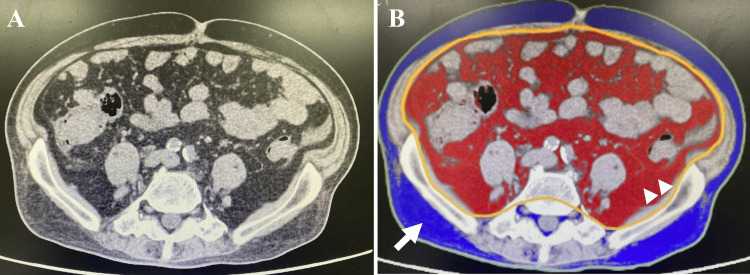
Abdominal CT slice at the level of the umbilicus, corresponding approximately to the L4 vertebral level, obtained upon admission. A: Plain CT scan at the level of the umbilicus. B: The visceral fat area was measured using a 320-row area detector CT scanner (CANON) with a slice thickness of 5 mm. The same plain CT scan at the level of the umbilicus, with visceral fat highlighted in red and subcutaneous fat highlighted in blue.The CT scan taken at the level of the umbilicus upon admission revealed a visceral fat area of 165 cm² (B, highlighted in red, indicated by arrow heads), indicating visceral fat accumulation. The subcutaneous fat area (B, highlighted in blue, indicated by arrow) was 109 cm².

Blood tests revealed pancytopenia; myelodysplastic syndrome was suspected by the hematology department, and the patient was observed. Other findings included elevated liver enzyme levels and mild inflammation, with a CRP level of 0.18 mg/dL. Regarding glucose metabolism, HbA1c was elevated at 8.1%, and the HOMA-IR was 4.1, indicating insulin resistance (cut-off value: ≤1.6). Body composition analysis using bioelectrical impedance analysis (BIA) showed a body fat mass of 25 kg, muscle mass of 40.4 kg, and a skeletal muscle index (SMI) of 6.6, suggesting reduced skeletal muscle mass. Although BIA provides only estimated values and may be less accurate in patients with neurological disorders such as CMT, it remains a useful and non-invasive tool for evaluating body composition and tracking longitudinal changes over time in routine clinical settings. A CT scan of the umbilical region revealed a visceral fat area of 165 cm² (Figure 1), indicating visceral fat accumulation.

**Figure 2:**
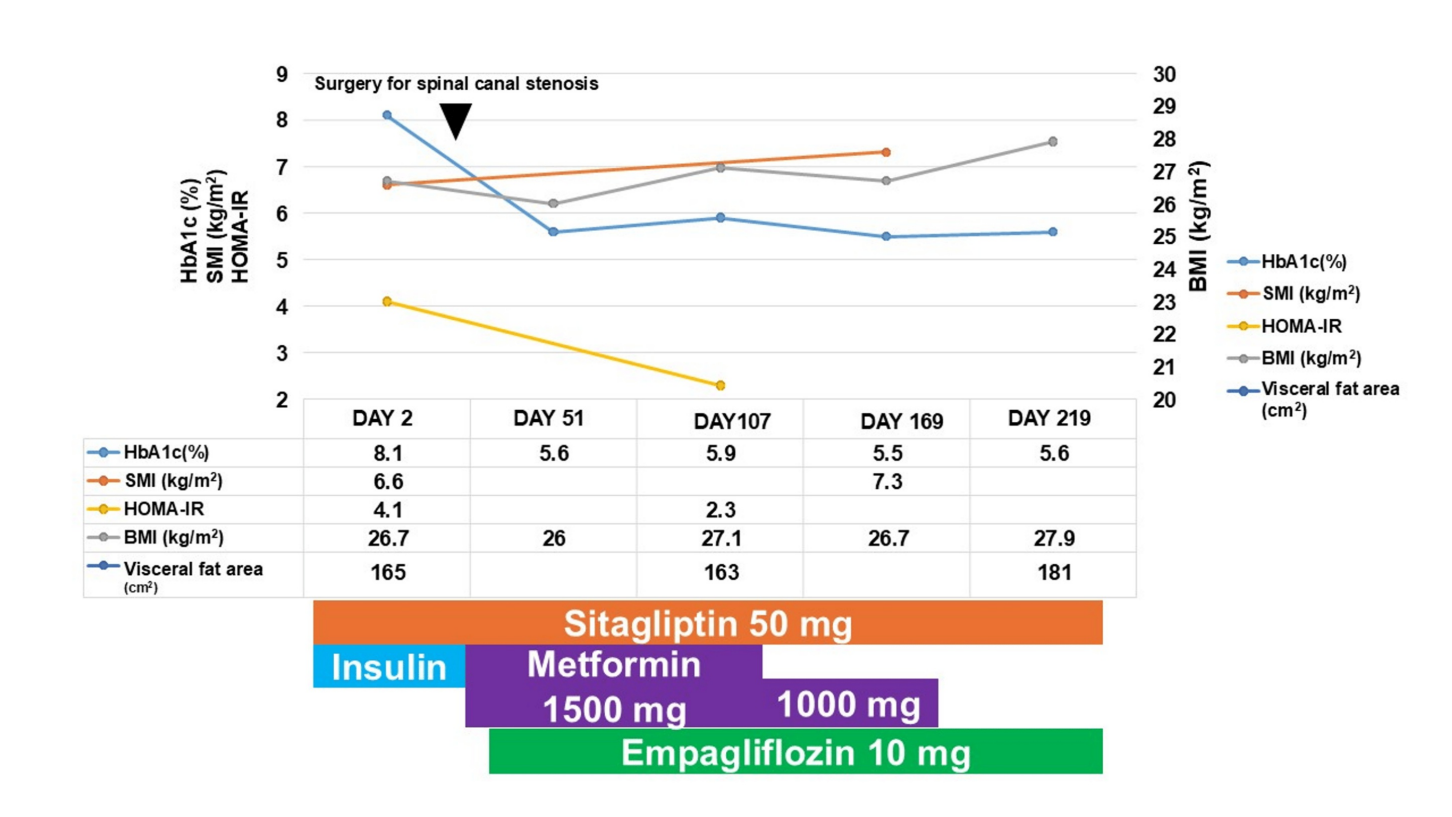
Clinical course of this case. Before surgery for lumbar spinal stenosis, blood glucose was managed with intensive insulin therapy and 50 mg of sitagliptin. After surgery, insulin was discontinued, and a combination therapy of metformin and 10 mg of empagliflozin was initiated. As a result, HbA1c significantly improved, and insulin resistance decreased. Although there was no marked change in visceral fat area in terms of body composition, an increase in skeletal muscle mass was observed. The downward arrowhead indicates the date of the spinal stenosis surgery.

Following admission, blood glucose management was initiated with sitagliptin (50 mg), and insulin therapy was intensified in preparation for surgery. Insulin glargine was administered at 12 units at bedtime, and insulin aspart was given before meals at doses of three units in the morning. Once glycemic control improved, surgery was performed for lumbar spinal canal stenosis. Postoperatively, insulin treatment was discontinued, and metformin was restarted. Due to visceral fat accumulation, empagliflozin 10 mg was initiated. While the BMI remained stable, the SMI improved to 7.3, and the HbA1c level improved to 5.5%. This improvement in SMI may be associated not only with the effects of the drug but also with muscle training, postoperative recovery, and enhanced activities of daily life (ADL). Metformin was discontinued, but the HbA1c level remained at 5.5% (Figure 2). 

## Discussion

We encountered a case of CMT complicated by diabetes mellitus. The main pathological condition of diabetes mellitus is insulin resistance due to the accumulation of visceral fat and decreased skeletal muscle mass, with preservation of endogenous insulin secretion. Treatment with the DPP-4 inhibitor sitagliptin and the SGLT2 inhibitor empagliflozin was associated with improvement in glycemic control.

CMT is a general term for peripheral nerve diseases caused by genetic mutations and was reported by Charcot, Marie, and Tooth in 1886 [6]. The main symptoms of CMT are muscle weakness and sensory disturbances, predominantly in the distal extremities of the limbs, due to peripheral neuropathy. CMT has various inheritance modes, including the autosomal dominant and recessive types. There was no difference in incidence between men and women. Onset usually occurs between 0 and 20 years of age, but in late‑onset forms, a secondary peak in diagnosis may be observed among individuals over 50 years of age [7]. Diagnosis was based on medical history, neurological examination, electrophysiological tests, and genetic testing. If CMT is suspected based on symptoms and neurological examination, a nerve conduction test is performed to examine the function of the peripheral nerves. In the diagnostic evaluation of CMT, procedures such as needle electromyography, nerve ultrasonography, and nerve biopsy (typically of the sural nerve near the ankle) may be performed in some cases. If abnormalities are found on these tests, a definitive diagnosis can be made using genetic testing [6]. In this case, CMT was diagnosed based on symptoms, neurological examination, and PMP22 mutation [8].

Numbness in the lower limbs is the earliest and most frequent complication of diabetic microangiopathy [9] and must be differentiated from neuropathic symptoms due to CMT. However, because muscle atrophy is observed in CMT, if muscle weakness or atrophy is observed in a patient with diabetes complaining of numbness in the lower limbs [10], it is necessary to suspect the coexistence of CMT, as in this case. It has also been reported that neurological disorders are further exacerbated when CMT is complicated by diabetes mellitus [11].

A total of 33 cases of CMT complicated by diabetes mellitus have been reported so far [5,11-16]. Most patients had type 2 diabetes mellitus [5]. Although the age at onset, severity, and specific characteristics of motor impairment vary depending on the subtype, all cases of comorbid diabetes mellitus reported to date have been CMT1. In CMT1, muscle weakness and sensory disturbances, mainly in the distal lower extremities, accompanied by gait disturbances, have been observed [17]. In patients with CMT, decreased lower limb skeletal muscle mass leads to reduced physical activity. This reduction in activity may result in visceral fat accumulation and skeletal muscle insulin resistance, both of which can contribute to the development of diabetes mellitus. However, CMT1 is the most frequently observed genetic mutation in CMT [17-19]; therefore, the coexistence rate of diabetes mellitus may also be high by chance.

There are few reports on the treatment of diabetes mellitus comorbid with CMT; however, metformin has been reported to improve glycemic control [20]. However, in this case, glycemic control was poor despite metformin administration. Although visceral fat accumulation was observed, TNF-α and IL-6 levels were within the normal range, suggesting that visceral fat-derived inflammatory cytokines may not have played a major role in this case. The insulin resistance may instead have been primarily related to reduced glucose uptake associated with decreased skeletal muscle mass, although this remains speculative and warrants further study in a larger cohort. In this case, decreased exercise owing to muscle atrophy caused by CMT and complications of spinal canal stenosis may have been involved in the worsening of blood glucose levels. Therefore, diet and exercise therapy were administered after admission, and insulin therapy, in addition to a DPP-4 inhibitor, was administered before surgery. After surgery, DPP-4 and SGLT2 inhibitors were administered in combination with exercise therapy.

In recent years, SGLT2 inhibitors have been used not only as therapeutic agents for diabetes mellitus but have also expanded their indications to include chronic kidney disease [21,22] and heart failure [23,24]. On the other hand, along with the decrease in visceral fat, a decrease in skeletal muscle, especially in the elderly, is a concern with SGLT2 inhibitor administration [25,26]. In this case, there were concerns regarding the exacerbation of decreased skeletal muscle mass and muscle weakness in the lower limbs. However, the SMI increased rather than decreased after administration. Recent meta-analyses on the effect of SGLT2 inhibitors on skeletal muscle mass have reported little effect; clinical studies in which empagliflozin was administered to elderly patients did not show any adverse effects on skeletal muscle mass in patients with a BMI of 23 or higher [27]. However, there have been reports of improved grip strength with SGLT2 inhibitors [28]. Regarding the combination therapy with sitagliptin, clinical studies have reported that patients in the DPP-4 inhibitor group were assigned to receive either SGLT2 inhibitors (ipragliflozin) or metformin. The combination of sitagliptin and SGLT2 inhibitors has been reported to reduce visceral fat compared to the combination of sitagliptin and metformin [29], with no difference in skeletal muscle mass [30]. The combination of DPP-4 and SGLT2 inhibitors may be a promising option for treating diabetes mellitus in patients with CMT and compromised muscle mass.

However, since this is a relatively short-term observation of a single case, it is necessary to accumulate more cases in the future to determine whether the combination of a DPP-4 inhibitor and an SGLT2 inhibitor is effective in the treatment of CMT complicated by diabetes mellitus. CMT is a progressive disease, and elevated blood glucose levels associated with diabetes mellitus may further reduce muscle mass through the action of two proteins, WWP1 and KLF15 [31]. Therefore, long-term glycemic control is important for maintaining patients’ ADL and quality of life (QOL).

## Conclusions

This case highlights the potential of combining a DPP-4 inhibitor (sitagliptin) with an SGLT2 inhibitor (empagliflozin), which may help improve glycemic control in a patient with CMT complicated by type 2 diabetes and appears not to have compromised skeletal muscle mass. This therapeutic approach may be particularly valuable for patients with reduced mobility and insulin resistance. Further studies are needed, but this strategy shows promise for preserving both metabolic health and physical function in this rare comorbidity.
